# Prognostic Value of Hepatocyte Growth Factor, Syndecan-1, and Osteopontin in Multiple Myeloma and Monoclonal Gammopathy of Undetermined Significance

**DOI:** 10.1100/2012/356128

**Published:** 2012-04-26

**Authors:** Jiri Minarik, Tomas Pika, Jaroslav Bacovsky, Pavla Petrova, Katerina Langova, Vlastimil Scudla

**Affiliations:** ^1^Department of Internal Medicine III, Palacky University and University Hospital Olomouc, 775 20 Olomouc, Czech Republic; ^2^Department of Clinical Biochemistry, Palacky University and University Hospital Olomouc, 775 20 Olomouc, Czech Republic; ^3^Department of Biophysics and Statistics, Palacky University of Olomouc, 775 20 Olomouc, Czech Republic

## Abstract

Our aim was to compare serum levels of selected biological parameters in different phases of multiple myeloma (MM) and monoclonal gammopathy of undetermined significance (MGUS) to determine their diagnostic and prognostic potential. A cohort of 234 individuals was assessed for serum levels of hepatocyte growth factor (HGF), syndecan-1/CD_138_ (SYN), and osteopontin (OPN). The patients with MM (*N* = 156) were divided into 3 groups: at the time of diagnosis (*N* = 45), in relapse/progression (*N* = 56), and in remission (*N* = 50). The analysis revealed significant differences of all three parameters in comparison of active and remission phase MM. Moreover, the parameters in active myeloma were significantly higher than in MGUS. Within the comparison of active disease (newly diagnosed and relapsing), there was no significant difference. Similar results were in remission phase MM and MGUS. There was no relationship of pretreatment levels of the parameters to therapeutic response. We conclude that serum levels of HGF, OPN, and SYN correspond to the activity of MM and might become useful in differentiation of MGUS, asymptomatic MM, and overt/symptomatic form of MM. The levels of all three parameters behave accordingly with MM activity. Pretreatment measurement without the assessment of their kinetics, however, has no relationship to therapeutic response.

## 1. Introduction

Multiple myeloma (MM) is a very heterogeneous disease with a different manifestation, course, and response to therapy. The biology of malignant cell as well as the microenvironment of the bone marrow account for such heterogeneity, offering novel targets for therapeutic approach. Several angiogenic cytokines, bone metabolism markers, cytoadhesive molecules, and other parameters with close relationship to the pathogenesis of MM were confirmed to have significant differences in monoclonal gammopathy of undetermined significance (MGUS) and in different phases of MM [1]. Some of them were identified as growth factors, cell cycle modificators, or even potent prognosticators whereas the role of many others is still not fully understood.

Based on recent findings that the serum levels of selected biological parameters might have a direct relationship to therapeutic outcome in patients with MM, we analyzed our historical cohort of patients in order to find convenient tools for diagnostic approach as well as for the risk stratification of patients with MGUS and MM.

Out of more than 20 biological parameters, we selected three with the highest rank of significant relationship to MM pathogenesis in previous studies [1–5]. These are the hepatocyte growth factor (HGF), syndecan-1 (SYN), and osteopontin (OPN).

The aim of our study was to estimate their diagnostic value in different phases of multiple myeloma and in MGUS, and to define whether their pretreatment levels can be used to predict therapeutic response.

## 2. Patients and Methods

We retrospectively analyzed a cohort of 234 individuals—156 patients with multiple myeloma and 78 patients with monoclonal gammopathy of undetermined significance (MGUS), who were assessed for serum levels of HGF, SYN, and OPN. The patients with MM were divided into three separate groups: at the time of diagnosis (*N* = 41), in relapse/progression (*N* = 65), and in remission (*N* = 50). Patients with active disease (i.e., at the time of diagnosis and in relapse/progression, *N* = 106) were then assessed for therapeutic response. Nonresponders (*N* = 54) were defined by achievement of less than 50% decrease of monoclonal immunoglobulin, and patients who responded (*N* = 52) had more than 50% reduction of monoclonal immunoglobulin. A subanalysis of 27 patients responding after autologous peripheral blood stem cell transplantation (ASCT) was carried out, defining also the differences between the serum levels of HGF, SYN, and OPN in patients achieving complete remission (CR) and very good partial remission (VGPR) according to IMWG criteria in comparison with patients who achieved partial remission (PR) only. In the rest of the patients who were treated by conventional chemotherapy without ASCT, the numbers of patients achieving PR and >PR were uneven, precluding a valid statistical analysis.

The MM patients were treated using high-dosed chemotherapy with support of autologous stem cell transplantation (31%, induction treatment using VAD regimen, V: vincristine, A: adriamycin, D: dexamethasone, conditioning with Mel200), thalidomide-based regimens (39%, regimens MPT and CTD, M: melphalan, P: prednisone, T: thalidomide, C: cyclophosphamide, D: dexamethasone), and a historical group using conventional chemotherapy (30%, regimens MP, VBMCP, and VAD, V: vincristine, B: BCNU, M: melphalan, C: cyclophosphamide, P: prednisone, A: adriamycin, D: dexamethasone).

Serum levels of hepatocyte growth factor (HGF), osteopontin (OPN), and syndecan-1/CD_138_ (SYN) were measured using the technique of quantitative sandwich enzyme immunoassay at the Department of Clinical Biochemistry. Levels of each parameter were then assessed within individual groups of MM and MGUS patients. In all patients, the assessment was carried out at the time of diagnosis, and in patients undergoing ASCT at the time of diagnosis and also on the day +100 after autologous transplantation. Finally, we compared the relationship of pretreatment serum levels of each parameter (HGF, OPN, and SYN) with therapeutic response.

The characteristics of the whole cohort are summarized in Table 1. Evaluation of cytogenetics was not performed in all the patients, and, therefore, the results were not included in the analysis.

The data were assessed by the tests of normality according to Shapiro-Wilk. It was found that the data do not have a normal distribution at *P* < 0.05; therefore, we used only nonparametric methods. For statistical estimation, we used Kruskal-Willis test, followed by multiple comparison with Mann-Whitney test with Bonferroni correction, and Wilcoxon test. The level of statistical significance of the Kruskal-Willis test was *P* < 0.05; in individual Mann-Whitney tests, the significance level after Bonferroni correction was *P* < 0.0083.

## 3. Results

The overall difference within the assessment of all three parameters (HGF, OPN, SYN) in MGUS and individual phases of MM course (i.e., at the time of diagnosis, in relapse/progression and in remission of the disease) was statistically significant (*P* < 0.0001). There were no significant differences between serum levels of HGF, SYN, and OPN within gender, Durie: Salmon (DS) staging (although there was a trend of higher levels of HGF and OPN in more advanced stages) or the International Stratification System (ISS).

**Figure 1:**
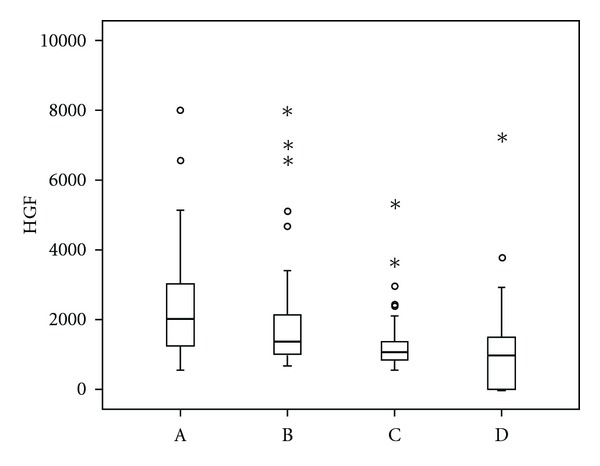
Comparison of serum levels of hepatocyte growth factor (HGF) in different phases of multiple myeloma and in monoclonal gammopathy of undetermined significance. Vertical axis: serum levels of hepatocyte growth factor (HGF, pg/mL). Horizontal axis: (A) multiple myeloma at the time of diagnosis, (B) multiple myeloma (MM) in relapse/progression, (C) remission phase multiple myeloma, (D) monoclonal gammopathy of undetermined significance (MGUS). The differences between active phase MM (A versus B) and remission phase myeloma and MGUS (C and D) were not statistically different. Significant differences were found between MM at the time of diagnosis and remission phase myeloma (A versus C; M 2001,0 versus 1049,0 pg/mL, *P* < 0,0001), MM at the time of diagnosis and MGUS (A versus D; 2001,0 versus 1228,0 pg/mL, *P* < 0,0001), MM in relapse/progression and remission phase MM (B versus C; M 1370,0 versus 1049,0 pg/mL, *P* < 0,0001), and MM in relapse/progression and MGUS (B versus D; 1370,0 versus 1228,0 pg/mL, *P* = 0,0002).

**Figure 2:**
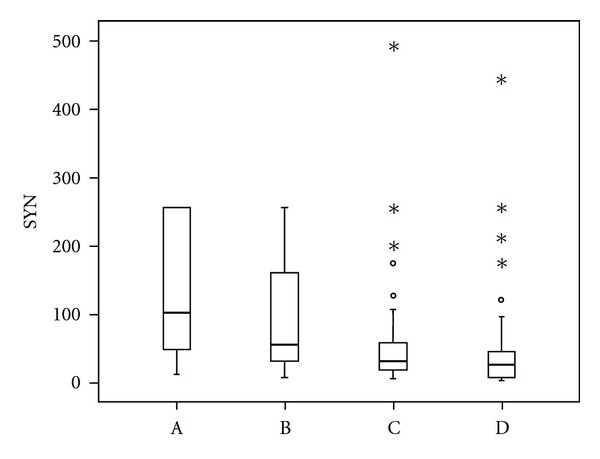
Comparison of serum levels of syndecan-1 (SYN) in different phases of multiple myeloma and in monoclonal gammopathy of undetermined significance. Vertical axis: serum levels of syndecan-1 (SYN, ng/mL). Horizontal axis: (A) multiple myeloma at the time of diagnosis, (B) multiple myeloma (MM) in relapse/progression, (C) remission phase multiple myeloma, (D) monoclonal gammopathy of undetermined significance (MGUS). The differences between active phase MM (A versus B), and remission phase myeloma and MGUS (C and D) were not statistically different. Significant differences were found between MM at the time of diagnosis and remission phase myeloma (A versus C; M 103,25 versus 31,06 ng/mL, *P* < 0,0001), MM at the time of diagnosis and MGUS (A versus D; M 103,25 versus 28,0 ng/mL, *P* < 0,0001), MM in relapse/progression and remission phase MM (B versus C; M 58,0 versus 31,06 ng/mL, *P* < 0,0001), and MM in relapse/progression and MGUS (B versus D; M 58,0 versus 28,0 ng/mL, *P* < 0,0001).

**Figure 3:**
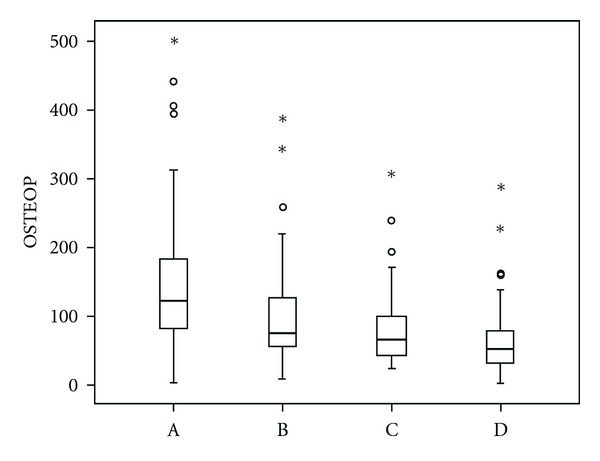
Comparison of serum levels of osteopontin (OPN) in different phases of multiple myeloma and in monoclonal gammopathy of undetermined significance. Vertical axis: serum levels of osteopontin (OPN, OSTEOP, ng/mL). Horizontal axis: (A) multiple myeloma at the time of diagnosis, (B) multiple myeloma (MM) in relapse/progression, (C) remission phase multiple myeloma, (D) monoclonal gammopathy of undetermined significance (MGUS). The differences between active phase MM (A versus B) and remission phase myeloma and MGUS (C and D) were not statistically different. The difference between MM in relapse/progression and remission phase MM was not significant, either. Significant differences were found between MM at the time of diagnosis and remission phase myeloma (A versus C; M 123,1 versus 66,55 ng/mL, *P* = 0,0003), MM at the time of diagnosis and MGUS (A versus D; M 123,1 versus 52,485 ng/mL, *P* < 0,0001), and MM in relapse/progression and MGUS (B versus D; M 74,975 versus 52,485 ng/mL, *P* = 0,0001).

Assessment of serum levels of HGF revealed statistically significant differences between medians (M) in patients with MM at diagnosis and in remission (M 2001,0 versus 1049,0 pg/mL, *P* < 0,0001), and in patients with MM in relapse/progression and in remission (M 1370,0 versus 1049,0 pg/mL, *P* < 0,0001), whereas the difference in HGF levels between patients at the time of diagnosis and in relapse/progression was not statistically significant (*P* = 0,051), Figure 1. Similar results showed the analysis of OPN and SYN. Within the assessment of serum levels of SYN, statistically significant differences of the medians were between the disease at the time of diagnosis and in remission (M 103,25 versus 31,06 ng/mL, *P* < 0,0001), and in relapse/progression versus remission of MM (M 58,0 versus 31,06 ng/mL, *P* < 0,0001), whereas the difference between newly diagnosed and progressing MM was not significant, showing just a trend (*P* = 0,041), Figure 2. Serum levels of OPN were significantly different only between MM at the time of diagnosis and in remission (M 123,1 versus 66,55 ng/mL, *P* = 0,0003). The levels of OPN between MM in relapse/progression and in remission (M 74,975 versus 66,55 ng/mL), and between MM at the time of diagnosis and in relapse/progression (M 123,1 versus 74,975) were not statistically significant (*P* = 0,101), Figure 3.

 All three parameters, that is, HGF, SYN, and OPN had significantly higher levels when comparing active MM (at the time of diagnosis and in relapse/progression) to MGUS. HGF had the following serum levels at the time of diagnosis, in relapse/progression and in MGUS: M 2001,0 and 1370,0 versus 1228,0 (*P* < 0,0001 and *P* = 0,0002). The levels of SYN were as follows: M 103,25 and 58,0 versus 28,0 (*P* < 0,0001 and *P* < 0,0001). The levels of OPN were M 123,1 and 74,975 versus 52,485 (*P* < 0,0001 and *P* = 0,0001). The levels of HGF, SYN, and OPN were not different when comparing remission phase MM to MGUS (*P* = 0,343).

The assessment of the predictive value of the three parameters revealed the following results: there was no statistically significant relationship between initial levels of any of the three molecules (HGF, SYN, and OPN) and the achievement of MM response. The same results were derived when comparing all patients with active disease and within the assessment of the subgroup of patients at the time of diagnosis and in relapse/progression of MM (Table 2). There was only a borderline significance for the prediction of therapeutic response based on the initial levels of SYN at the time of diagnosis.

In the subanalysis of patients responding after ASCT, the results were similar regardless of the depth of therapeutic response. Patients achieving >PR had significantly lower levels of the three parameters (HGF, SYN, and OPN) in comparison with initial measurement (*P* = 0,0003, *P* = 0,005, and *P* = 0,033 resp.).

## 4. Discussion

Angiogenesis plays a key role in MM pathogenesis [6, 7]. Vacca et al. have first described markedly increased angiogenesis in MM in comparison with MGUS [8]. Since then, it has been intensively studied and correlated to plasma cell proliferation as well as to the malignant transformation [9]. Some studies have shown that angiogenesis has a prognostic value in MM [9, 10]. It affects the pathogenesis in two ways: the newly formed blood vessels supply the tumor with oxygen and nutrients, and angiogenesis modulates the interaction of myeloma plasmocytes with stromal cells by paracrine stimulation of tumor cell migration and proliferation [9].

Among all potential angiogenic cytokines, hepatocyte growth factor has been acknowledged as the pivotal factor involved in multiple myeloma angiogenesis. HGF is mainly produced by bone marrow. It promotes cell growth, invasion, motility, and neovascularisation via its receptor, c-MET, which is highly expressed in epithelial cells and also in MM plasmocytes [11, 12]. In the context of multiple myeloma, HGF was found to have an impact on bone disease, too. It promotes the formation of osteoclasts from hematopoetic precursor cells, attracts them to sites of bone resorption, and also inhibits bone morphogenic protein (BMP)—induced osteoblastogenesis [13–15]. On the other hand, its concentrations vary during bone marrow harvesting which might account for conflicting results in some MM studies [16]. The concentrations of HGF in peripheral blood are less likely affected by the technique of harvesting and should therefore better correspond to the activity of the disease [2]. Moreover, positive correlation between serum levels of HGF and bone marrow microvessel density was reported [3].

Patients with multiple myeloma have significantly higher levels of HGF than healthy controls [17]. The behavior of hepatocyte growth factor levels after treatment has been already documented by several reports [17, 18]. In accordance with our findings, the authors describe a decrease in HGF levels with treatment response, and an increase during the disease relapse. Our paper in addition compares serum levels of HGF in the most prominent phases of multiple myeloma, that is, at the time of diagnosis, in remission and in relapse/progression of the disease in comparison with MGUS. Possible role of HGF as an auxiliary diagnostic and prognostic tool documented by significant differences between active disease and premalignant MGUS or remission phase myeloma is, however, attenuated by certain overlapping of marginal values in all the groups. Nevertheless, the decrease of HGF after treatment expects a relationship of HGF to the activity of the disease, suggesting its application in the assessment of therapeutic response and/or the evaluation of the disease progression.

Our findings oppose to the studies of Pour, and Svachova et al. who suggested the role of HGF as a predictive factor for MM treatment response using ASCT, conventional therapy, and thalidomide or bortezomib-based regimens [19, 20]. Having a larger cohort, our study did not unambiguously find significant differences in therapeutic outcomes based on HGF levels in any treatment arm. According to our findings, the levels of HGF more likely correspond to the activity of the disease and are higher in more advanced stages of MM, similarly as in the study of Alexandrakis et al. [2]. Together with the limitations of a small group statistics, correspondence of HGF levels to the extent of MM might be the reason for the poor outcome in patients with high levels of HGF treated in the mentioned studies.

Osteopontin is a glycoprotein involved in angiogenesis, adhesion, cell proliferation, and apoptosis [21]. It is produced by many cell types such as lymphocytes, osteoclasts, or endothelial cells. Several studies described elevated serum levels of OPN in multiple myeloma, in comparison with both normal subjects as well as MGUS individuals [4, 22]. OPN is produced by osteoclasts and especially directly by plasma cells, and it is thought to stimulate tumor proliferation together with proangiogenic effect on blood vessels, endothelial cell survival, and migration [21]. High expression of OPN inversely correlates with myeloma bone disease [23].

A very recent paper by Sfiridaki et al. describes the role of OPN in MM bone destruction and angiogenesis [24]. In accordance with our findings, the serum levels of OPN correlated with the activity of the disease and decreased after treatment. Our findings contribute by adding the relationship of OPN serum levels behavior and therapeutic outcome. Nevertheless, similarly as in the case of HGF, pretreatment levels of OPN had no impact on the final effect of the therapy. Interestingly, OPN did not correlate with HGF, suggesting an effect on different pathways in the regulation of both, angiogenesis, and myeloma proliferation regulation.

Syndecan-1/CD_138_ is a transmembrane proteoglycan expressed by most myeloma plasma cells [25]. Its role in MM includes the regulation of adhesion, migration, and promotion of proliferation by mediating the effects of growth factors in plasma cells. Recent papers have concedeed also its role in endothelial invasion and angiogenesis [26]. Similarly as some other membrane proteins, it can be cleaved from the cell surface and released into the extracellular and later intravascular compartment. High serum levels of soluble syndecan-1 have been acknowledged as an indicator of poor prognosis [27]. Our previous studies confirmed the role of SYN in the transformation of MGUS into MM and its superiority among several other bioactive molecules in prognostication of MM overall survival in the era of conventional therapy [1, 28]. The presented study showed the limitations for the utilization of SYN as a robust prognosticator. Despite its significant differences between active MM and MGUS, and even between active and remission phase MM, the levels of SYN themselves were not capable of the distinction between the different phases of the disease. Marginal values in all the groups were overlapping, disabling a clear identification of individual phases. Similarly as in the case of HGF or OPN, serum levels of SYN correlated with the activity of MM rather than with its future outcome.

Our results support the role of hepatocyte growth factor, syndecan, and osteopontin in the transformation of MGUS into MM and in the activity of MM. It is not, however, evident, whether these differences contribute primarily to the evolution of symptomatic disease or whether they are the result of multiple myeloma effect on the bone marrow microenvironment. Nevertheless, the absolute levels of these parameters could become convenient auxiliary tools for the differentiation of MM and MGUS, and especially in the setting of initial (asymptomatic, smoldering) MM. Similarly, significant decrease of HGF, SYN, and OPN in the course of the disease corresponds to the disease activity and might be helpful in the assessment of therapeutic response as well as in the choice of individualized approach.

On the other hand, we have not confirmed the predictive role of any of these parameters for the achievement of therapeutic response in any of the regimens used (conventional chemotherapy, high-dosed chemotherapy with support of ASCT, and thalidomide-based regimens). We therefore conclude that the serum levels of HGF, SYN, and OPN are likely to reflect the activity of MM rather than its aggressiveness or resistance to therapy.

## Figures and Tables

**Table 1:** Characteristics of the analyzed group.

	MM	MGUS
*N*	156	78
Age median (years)	65 (range 32–86)	62 (range 31–86)
M/F ratio	1,08 : 1	0,56 : 1
MIg type		
IgG	98 (63%)	60 (77%)
IgA	43 (27%)	14 (18%)
Bence-Jones	14 (9%)	3 (4%)
IgD	1 (1%)	—
Biclonal	—	1
*κ*/*λ* ratio	2,55	1,7

MM stage		
ISS 1	98 (63%)	—
ISS 2	39 (25%)	—
ISS 3	19 (12%)	—
DS I	15 (10%)	—
DS II	71 (46%)	—
DS III	70 (44%)	—
Substage A	149 (96%)	—
Substage B	7 (4%)	—

**Table 2:** Differences of pretreatment serum levels of hepatocyte growth factor, syndecan-1, and osteopontin in multiple myeloma treatment responders and nonresponders.

	Treatment responders (median)	Treatment nonresponders (median)	*P*-value
HGF diagnosis	2139,0	1627,0	0,114
HGF relapse	1365,0	1549,0	0,958
SYN diagnosis	151,0	46,2	**0,041**
SYN relapse	46,9	78,6	0,589
OPN diagnosis	131,5	110,8	0,448
OPN relapse	60,3	76,4	0,133

HGF: hepatocyte growth factor (pg/mL), SYN: syndecan-1 (ng/mL), OPN: osteopontin (ng/mL). Treatment responders: patients achieving ≥ partial remission after treatment; nonresponders: patients who did not reach partial remission and/or were progressing after treatment.

Statistical estimation was done using Mann-Whitney test at *P* < 0,05. Only the pretreatment serum levels of soluble syndecan-1 showed borderline significant differences in comparison of treatment responders and nonresponders.
